# A confounding bronchopulmonary situs abnormality after bilateral lung transplantation

**DOI:** 10.1093/jscr/rjac107

**Published:** 2022-04-23

**Authors:** Subha Ghosh, Steven Fox, Atul C Mehta

**Affiliations:** Thoracic Imaging, Imaging Institute, Cleveland Clinic, Cleveland, OH, USA; Respiratory Institute, Cleveland Clinic Foundation, Cleveland Clinic, Cleveland, OH, USA; Respiratory Institute, Cleveland Clinic Foundation, Cleveland Clinic, Cleveland, OH, USA

## Abstract

A 36-year-old female with a past medical history of primary ciliary dyskinesia and bilateral lung transplantation was noted to have a rare and confounding postsurgical anatomy acquired as a result of transplantation. Bronchoscopy and computed tomography showed isomeric main bronchi with a tri-lobed right lung, a bi-lobed left lung and dextrocardia. This rare phenomenon can be observed in lung transplant recipients with situs ambiguous morphology of their native lungs, who receive donor lungs with normal situs solitus morphology. To the best of our knowledge, ours is the first reported case of such a composite bronchopulmonary situs abnormality. Careful review of bronchial anatomy should be done with the help of CT imaging prior to undertaking any bronchial interventions in these subset of patients with bronchial isomerism and bilateral lung transplantation.

## INTRODUCTION

Main bronchial isomerism with normal anatomy of the lungs has not been previously reported. This can rarely occur in lung transplant recipients with situs ambiguous morphology of their native lungs, who receive donor lungs with normal situs solitus morphology. In such patients, careful review of anatomy should be done with the help of computed tomography (CT) imaging prior to undertaking any endobronchial interventions.

## CASE REPORT

**Figure 1:**
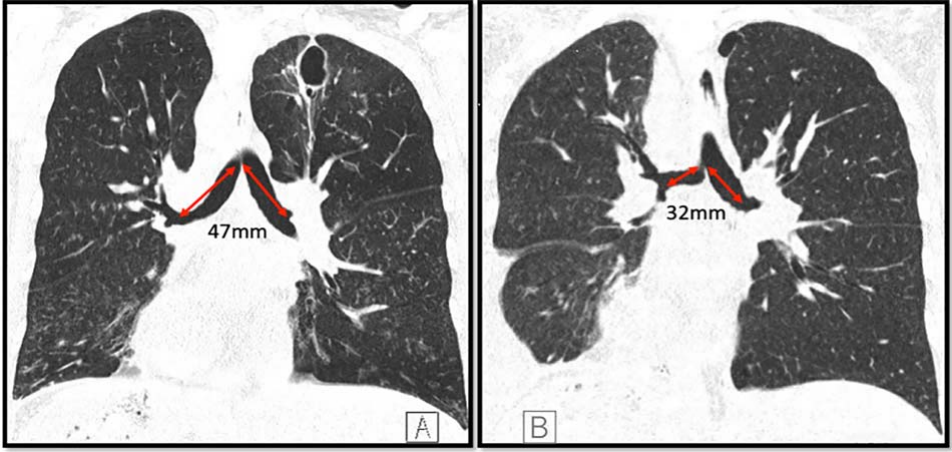
Coronal CT images depicting both main bronchi with the length of 47 mm prior to the lung transplantation (**A**); length of both main bronchi post transplantation is ~32 mm; the right main bronchus is now at an approximate right angle with the trachea (**B**); note the presence of dextrocardia in both images and a thin-walled postinfectious left upper lobe cavity in the native lung.

**Figure 2:**
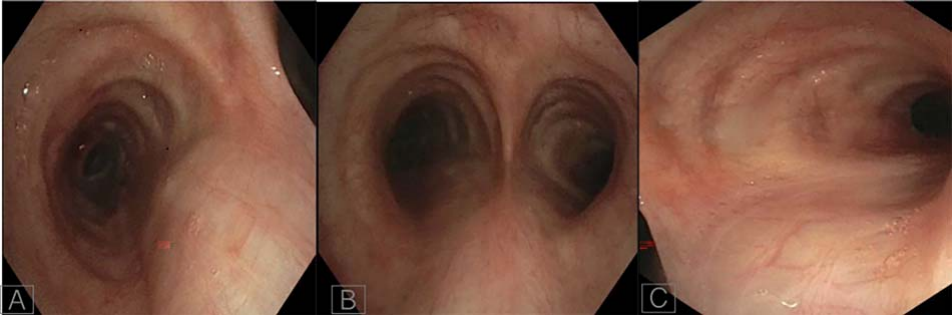
Bronchoscopy images of left main bronchus (**A**), carina (**B**) and right main bronchus post transplantation (**C**).

A 36-year-old female with past medical history of primary ciliary dyskinesia (PCD), chronic sinusitis and bronchiectasis with *Pseudomonas Aeruginosa* and Aspergillus colonization underwent bilateral lung transplantation. Based on the CT of the chest, both right and left main bronchi prior to lung transplantation were 47 mm in length, with a carinal bifurcation angle of ~55° (Fig. 1A). Prior to lung transplantation she had bi-lobed right and left lungs, with a right main bronchus which was as long as the left, which is a feature of situs ambiguous, i.e. left isomerism (bilateral left-sidedness). She had normal atrio-ventricular and ventriculo-arterial morphology though her cardiac apex was mildly shifted to the right (dextrocardia). Post transplantation, she was admitted as an outpatient for evaluation of declining lung function as detected on pulmonary function tests. She underwent repeat CT and bronchoscopy with transbronchial biopsies to evaluate for graft rejection. Interestingly, after transplantation her repeat CT (Fig. 1B) and surveillance bronchoscopy (Fig. 2A–C) revealed both right and left main bronchi that were 32 mm in length, with a normal tri-lobed right lung and a bi-lobed left lung. The right main bronchus was now at a right angle (90°) with the trachea. The composite bronchopulmonary situs was the result of isomeric recipient main bronchi (situs ambiguous) and normal donor lungs (situs solitus).

## DISCUSSION

End-stage PCD with bronchiectasis accounted for <4% of bilateral lung transplantation performed between January 1995 and June 2018 [1]. In a study by Shapiro *et al*. [2] >50% of all PCD patients also had situs abnormalities. Kartagener’s syndrome (KS), a PCD variant characterized by a clinical triad of situs inversus, bronchiectasis and chronic sinusitis, was seen in 41% PCD patients, while 12% had situs ambiguous along with laterality defects. The most challenging part of treating end-stage PCD and variants such as KS with bilateral lung transplantation is its complex anatomy [3].

Situs solitus is defined as the usual or normal arrangement of internal organs along the left–right axis. Situs inversus is defined as the complete mirror arrangement of internal organs along the left–right axis. Situs ambiguus therefore ranges between the spectrum of solitus and inversus, in which the internal organs neither have the normal or usual nor the mirror-imaged arrangements [4, 5]. Thus, in situs ambiguous, there can be either bilateral left sidedness (left isomerism/hyparterial bronchi) or bilateral right sidedness (right isomerism/eparterial bronchi) [5]. In situs solitus (normal) the right main bronchus has an average length of 20 mm before it bifurcates into right upper lobe bronchus and bronchus intermedius. The left main bronchus is narrower and longer than its counterpart with an average length of 50 mm [6].

In our patient, the lengths of the right and left main bronchi prior to lung transplantation were 47 mm each, which was suggestive of left-sided isomerism. Notably, her post transplantation CT (Fig. 1B) and bronchoscopy (Fig. 2A–C) revealed both main bronchi to be of equal length (32 mm), which was longer than a normal right-sided bronchus (20 mm) yet shorter than a normal left-sided bronchus (50 mm). The right main bronchus was now at a right angle with the trachea and connected to a normal tri-lobed donor lung, while the left bronchus was anastomosed to a normal bi-lobed donor lung.

During lung transplantation the bronchial anastomosis is kept close to the bifurcation of the main bronchus to allow retrograde revascularization via bronchial arteries and to reduce risk of ischemic complications [3]. Thus, our patient with left-sided bronchial isomerism ended up with intermediate equal-length main-stem bronchi to facilitate surgical bronchial anastomosis with normal donor lungs (Fig. 1B). The histopathological samples obtained during the biopsy showed no evidence of rejection. Patient was discharged in stable condition to follow-up with her transplant pulmonologist.

To our knowledge, this is the first reported case of such a rare composite bronchopulmonary situs abnormality. This case highlights the clinical importance of screening for bronchial isomerism in transplant patients. All transplant physicians and interventional pulmonologists should be aware of this unusual anatomy before undertaking diagnostic or therapeutic endobronchial interventions, such as bronchoscopic interventions or stent placement in post-transplant strictures.
